# Plasma concentrations of IL-6, MIP-1β, IP-10, and PTX-3 as predictors of the immunological response to antiretroviral treatment in people with HIV

**DOI:** 10.3389/fimmu.2024.1447926

**Published:** 2024-08-29

**Authors:** Marta Mejías-Trueba, Abraham Saborido-Alconchel, Ana Serna-Gallego, María Trujillo-Rodríguez, Esperanza Muñoz-Muela, Silvia Llaves-Flores, Nuria Espinosa, Cristina Roca-Oporto, Marta Herrero, Cesar Sotomayor, Luis F. López-Cortes

**Affiliations:** ^1^ Department of Pharmacy, University Hospital Virgen del Rocío, Seville, Spain; ^2^ Clinical Unit of Infectious Diseases, Clinical Microbiology and Parasitology. Institute of Biomedicine of Seville/Virgen del Rocio University Hospital/CSIC/University of Seville, Seville, Spain

**Keywords:** HIV infection, IL-6, IP-10, MIP-1β, PTX-3, immunologic response

## Abstract

Despite effective antiretroviral therapy (ART), 15-30% of people with HIV experience poor CD4^+^ T-cell recovery, termed immunologic non-responders (INR). This study aims to evaluate whether pre-ART plasma levels of interleukin-6 (IL-6), interferon gamma-induced protein-10 (IP-10), macrophage inflammatory protein-1-β (MIP-1β), and/or pentraxin-3 (PTX-3) could predict subsequent immunologic recovery. Seventy-four participants were enrolled and classified as INR and immunologic responders (IR) based on CD4^+^/CD8^+^ ratio increase over 24 months after starting ART. The results showed no significant differences in cytokine levels between INR and IR. Therefore, IL-6, IP-10, MIP-1β, and PTX-3 were unsuitable as predictive markers of poor immune recovery.

## Background

Since the introduction of triple therapy as the gold standard of antiretroviral therapy (ART), 15-30% of people with HIV (PWH) experience a limited increase in CD4^+^ T lymphocyte count despite sustained virological suppression. This phenomenon is more common in individuals with a low nadir of CD4^+^ T cells, older age, and coinfections, often referred to in the literature as immunological non-responders (INR) (1). Although its definition lacks consensus, INR has historically been defined based on the increase of absolute CD4+ T lymphocyte counts (aCD4) above various thresholds within a specific timeframe (2). However, aCD4 values can exhibit significant variations within the same patient due to fluctuations in total white blood cell counts, lymphocyte subset values, and test imprecisions (3). In comparison, the absolute CD4+/CD8+ ratio in untreated HIV-infected patients is less prone to variations in repeated measurements, and its prognostic value is similar to aCD4 (3–5).

In PWH and receiving ART, the maintenance of CD4+ cell counts involves a dynamic balance between *de novo* production, CD4+ cell destruction, and trafficking to and from lymphoid organs. Several mechanisms have been suggested to underlie this phenomenon (2, 6); however, none explain it by itself, being highly likely that several of the proposed mechanisms could provide feedback on each other, leading to an uncontrolled vicious circle.

From a clinical perspective, a higher incidence of serious non-AIDS events (SNAEs) such as cardiovascular diseases, neurocognitive disorders, malignancies, and metabolic diseases have been observed in INR compared to those who achieve a good immune recovery, known as immunological responders (IR) (7, 8).

Consequently, numerous studies have investigated factors that may contribute to an INR response after varying durations of ART. Some studies have identified predictive markers, such as elevated plasma concentrations of interleukin 6 (IL-6) (9), macrophage inflammatory protein 1-β (MIP-1β) (10), and pentraxin 3 (PTX-3) (11), in patients assessed before initiating ART. Furthermore, Interferon-γ (IFN-γ)-induced protein 10 (IP-10 or CXCL-10) is a chemokine associated with immune cell trafficking to inflammatory sites. In the context of HIV infection, IP-10 plasma levels are typically elevated in most HIV-infected individuals. This chemokine has consistently shown an association with HIV disease progression (based on CD4+ counts) during the acute HIV infection phase (12). Notably, in a previous cross-sectional study within our cohort (comprising INR, 67 individuals; immunological responders (IR), 37 individuals; and healthy controls, 33), IL-6 and IP-10 were the only markers found to have higher concentrations in the INR group compared to the other two groups (unpublished data).

Given its clinical implications, this study aimed to assess whether a limited set of plasma inflammatory markers measured before starting ART can predict subsequent poor immunological recovery.

## Material and methods

### Setting and study design

This retrospective observational study included adults PWH diagnosed at the Virgen del Rocío University Hospital in Seville, Spain, between March 2017 and March 2020. It was designed and carried out according to the Declaration of Helsinki and the principles of Good Clinical Practice and approved by the Committee on the Ethics of Research on Medicinal Products of Seville (UCE -VIH -1-2023).

### Participants, follow-up, and end-points

As previously mentioned, since the CD4+/CD8+ ratio is less prone to variations in repeated measurements and has a prognostic value similar to that of aCD4 (3–5, 13), we used the CD4+/CD8+ ratio instead of aCD4 to evaluate immunological recovery.

Eligible participants were PWH with a baseline CD4^+^/CD8^+^ ratio <0.65, who maintained an undetectable viral load in >95% of the determinations (single blips were admitted) during a follow-up of at least two years and with available plasma sample just before starting ART. After 24 months on ART, we considered INR those whose CD4^+^/CD8^+^ ratio increased ≤0.3 and IR if the ratio increased >0.6. We selected these cut-off points based on a previous study by our (3). Thus, the primary end-point was to assess whether IL-6, IP-10, MIP-1β, and/or PTX-3 plasma levels before starting ART could predict subsequent immunological recovery.

### Quantification and analysis of plasma inflammatory markers

Plasma was aliquoted into cryotubes and stored at -80°C until subsequent assays. IL-6, IP-10, and MIP-1β were measured using a multiplex bead-based immunoassay (MILLIPLEX^®^; Merck EMD Millipore, Billerica, MA) following the manufacturer’s instructions. PTX-3 was measured by an enzyme-linked immunosorbent assay [Human Pentraxin 3/PTX-3 ELISA Kit (A73792)] according to the manufacturer’s instructions. All samples were analyzed in duplicate and repeated when the coefficient of variation (CV) was greater than 30%.

### Statistical analyses

Descriptive statistics were used for demographic, epidemiological, and clinical data. The results were expressed as medians and interquartile ranges (IQR) for quantitative variables, number of cases and percentages for categorical variables, and proportions with 95% confidence interval (CI_95%_). Comparisons between groups were evaluated using the χ^2^ and the Mann–Whitney U tests. The Spearman rank correlation coefficient (ρ) were used to assess correlations between variables.

The predictive values of the plasma concentrations of IL-6, MIP-1β, IP-10, and PTX-3 for predicting immune recovery at 24 months of follow-up were evaluated by receiver operating characteristic curves (ROC). The points estimated on the ROC curve whose sensitivity and specificity gave the maximal Youden’s index were considered the optimal cut-off, and the corresponding sensitivity, specificity, and area under the curve (AUC) were chosen if the AUC was different from 0.5 (null: AUC = 0.5). Likewise, the diagnostic odds ratio (OR) was calculated as a single indicator of test performance that does not depend on prevalence (14).

For the categorical dependent variable (INR *vs*. IR), a multivariate logistic regression analysis was performed, including the baseline variables gender, age, basal CD4^+^/CD8^+^, viral load, presence of viral hepatitis, and plasma levels of IL-6, PTX-3, IP-10, and MIP-1β. Statistical analyses were performed with IBM software (SPSS, version 25.0; SPSS, Chicago, IL). Graphs were generated with GraphPad Prism Software, v.9.0.0. P-values <0.05 were considered significant.

## Results

### Characteristics of the participants

The study included 74 treatment-naïve PWH with a median age at diagnosis of 35 years (28–46); 64 participants (86.5%) were male, of whom 54 (73%) were men who have sex with men (MSM). Forty-one were classified as INR, and the remaining 33 as IR. The median follow-up of the overall cohort was 4.4 years (IQR, 3.5-5.3) years. Their baseline demographic and clinical characteristics are summarized in **Table 1**.

**Table 1:** Baseline and evolutionary characteristics (month 24) of study patients.

	IR (n= 33)	INR (n= 41)	p
**Male sex**, n (%)	27 (81.8)	37 (90.2)	0.295
**Age**, years	36 (26–46)	34 (29–44)	0.683
**BMI**, kg/m^2^	41.6 (37.2–43.9)	42.1 (39.7–45.5)	0.360
Risk for HIV infection, n (%)			
MSM	24 (72.7)	30 (73.2)	0.983
HTX	9 (27.3)	10 (24.4)	
Others	–	1 (2.4)	
Previous to ART			
**HIV-RNA**, copies/mL	200000 (56300–495500)	164000 (38200–39200)	0.287
**HIV-RNA >**100000 copies/mL	20 (60.6)	25 (61.0)	0.974
**CD4^+^ T-cell count**, cells/μL	337 (206–444)	207 (96–319)	**0.006**
0**–**200	7 (21.2)	19 (46.3)	
201**–**350	11 (33.3)	13 (31.7)	
351**–**500	9 (27.3)	4 (9.8)	
>500	6 (18.2)	5 (12.2)	
**CD4^+^/CD8^+^ ratio**	0.37 (0.21–0.52)	0.34 (0.13–0.47)	0.507
0.01–0.15	11 (33.3)	4 (9.7)	
0.16–0.30	8 (24.2)	9 (21.9)	
0.31–0.45	13 (39.4)	11 (26.8)	
0.46–0.64	11 (33.3)	11 (26.8)	
ART regimen			
InSTI + 2NRTI	29 (87.9)	37 (90.2)	0.397
InSTI + 1NRTI	4 (12.1)	4 (9.7)	
**CD4^+^/CD8^+^ ratio**, month 24	1.19 (0.99-1.37)	0.53 (0.35-0.62)	**< 0.001**
**Δ CD4^+^/CD8^+^ ratio**, month 24	0.80 (0.71-0.93)	0.16 (0.13-0.24)	**< 0.001**
**CD4+ T cell count month 24,** cells/μL	687 (543–865)	523 (381–662)	**0.001**

Data are expressed as median (interquartile range) or n (%). IR, immune responder. INR, immunological non-responders. BMI, body mass index. MSM, men who have sex with men. HTX, heterosexual. Significant p values are displayed in bold.

None were co-infected with hepatitis C virus (HCV), while two participants in the INR group had positive HBsAg tests. All participants started and maintained ART with an integrase inhibitor (INI)-based regimen plus one or two nucleos(t)ide reverse transcriptase inhibitors (NRTIs), p = 0.397 for IR *vs*. INR.

The baseline CD4^+^/CD8^+^ ratio was similar between IR [0.37 (0.21–0.52)] and INR [0.34 (0.13–0.47)], p = 0.507. As expected, after 24 months on suppressive ART, the increases were quite different [IR, 0.80 (0.71-0.93) *vs*. INR, 0.16 (0.13-0.24), p < 0.001].

### Predictive values of plasma soluble inflammatory markers for CD4^+^/CD8^+^ ratio recovery

**Figure 1:**
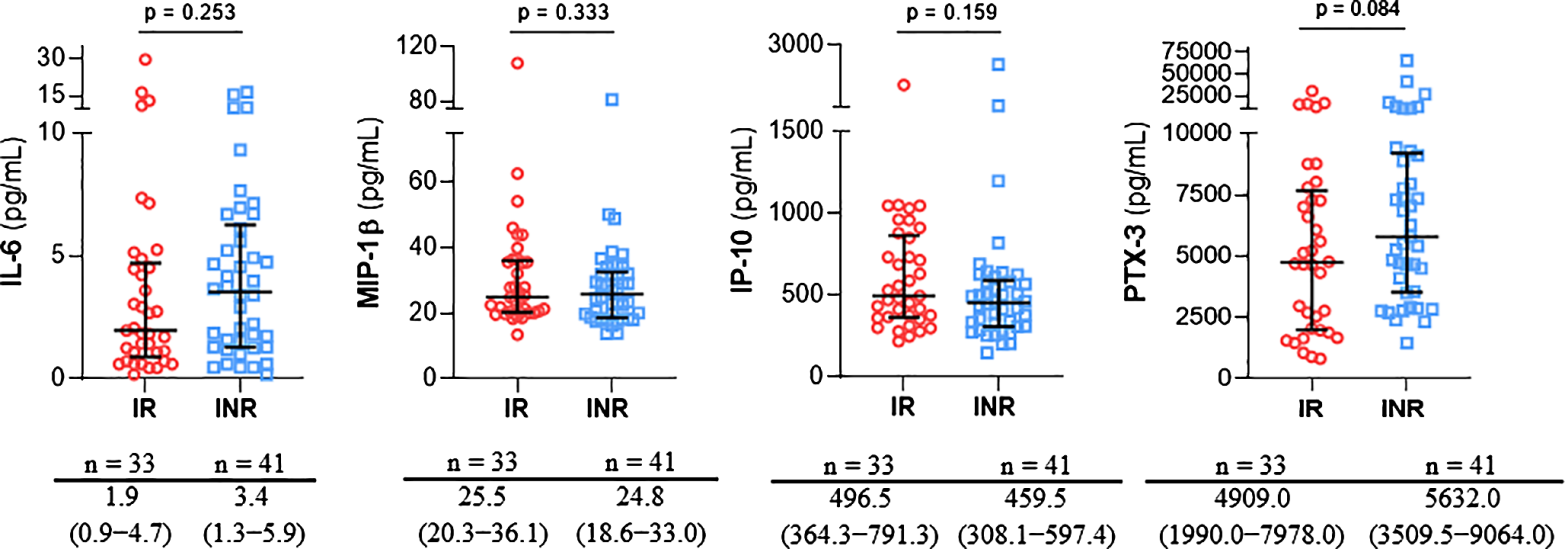
Comparison of medians and interquartile ranges (IQR) of IL-6, MIP-1β, IP-10 and PTX-3 plasma levels in immunological responders (IR) and immunological non-reponders (INR) based on subsequent immune recovery.

Plasma concentrations of the selected inflammatory markers in both groups (IR and INR) before starting ART are shown in **Figure 1**, without significant differences between them. There were weakly correlations between IL-6 and PTX-3 (ρ = 0.248, p = 0.018) and MIP-1β (ρ = 0.227, p = 0.026), and between IP-10 and MIP-1β (ρ = 0.385, p < 0.001).

We performed receiver operating characteristic (ROC) analyses to assess their predictive values for a poor immune recovery (**Table 2**). The resultant AUC for all of them was low. However, the diagnostic odds ratio for PTX-3 levels ≥ 2173.5 pg/ml and the combination of PTX-3 levels ≥ 2173.5 pg/ml and IP-10 <665.1 pg/ml were 15.2 (CI_95_, 1.8–128.3) and 7.3 (2.4–22.2), respectively; i.e., the odds of a positive result among INR was 15 and 7 times higher than among IR. The analysis of other combined predictive effect of multiple factors does not improve the results.

**Table 2:** ROC curve analysis of IL-6, PTX-3, IP-10, MIP-1β and combination PTX-3 + IP-10.

	AUC	Cut-off	SE (%) (CI_95_)	SP (%) (CI_95_)	PPV (%) (CI_95_)	NPV (%) (CI_95_)	Diagnostic OR (CI_95_)
**IL-6**	0.578	**≥**3.225	53.7 (38.7-67.9)	63.6 (46.6-77.8)	64.7 (47.9-78.5)	52.5 (35.5-67.1)	2.03 (0.79- 5.18)
**PTX-3**	0.597	**≥**2173.5	97.5 (87.1-99.6)	28.1 (15.6-45.4)	62.9 (50.5- 73.8)	90.0 (59.6- 98.2)	15.2 (1.8- 128.3)
**IP-10**	0.596	**<**665.1	87.8 (74.5-94.7)	36.4 (22.2-53.4)	63.2 (50.2-74.5)	70.6 (46.9-86.7)	4.11 (1.27- 13.31)
**MIP-1β**	0.566	**<**18.4	24.4 (13.8-39.3)	93.9 (80.4-98.3)	83.3 (55.2- 95.3)	50.0 (37.9-62.1)	5.00 (1.01-24.71)
**PTX-3 + IP-10**	0.706	**>**0.5	85.0 (70.9-92.9)	56.3 (39.3-71.8)	70.8 (56.8-81.8)	75.0 (55.1-88.0)	7.3 (2.4-22.2)

AUC, area under the curve; SE, sensibility; SP, specificity; PPV, positive predictive value; NPV, negative predictive value.

To identify which variables were independently associated with CD4^+^/CD8^+^ ratio recovery, multivariate logistic regression analysis (INR *vs.* IR) was performed. Accordingly, only PTX-3 levels ≥ 2173.5 pg/ml [OR, 16.5 (CI_95_, 1.9-143.9)] and IP-10 <665.1 pg/ml [OR, 4.5 (CI_95_, 1.3-15.7)] were independently associated with a poor immune recovery (INR).

## Discussion

Our results suggest that IL-6, IP-10, MIP-1β, and PTX-3 lack sufficient power to predict a subsequent poor immune recovery. Similar levels of these cytokines were observed in IR and INR. ROC curve analyses (**Table 2**) demonstrated low sensitivity, specificity, and predictive values for these biomarkers. Specifically, PTX-3 exhibited a good sensitivity (97.5%), but its specificity was notably low. Conversely, MIP-1β displayed high specificity (93.9%) but low sensitivity (24%). Therefore, these indicators are unsuitable as predictive markers for immune recovery in clinical practice.

To date, few studies have highlighted the role of cytokines in HIV infection and have analyzed their utility as potential predictive markers of disease progression or immune restoration. However, their results are sometimes contradictory. In this context, our study aimed to evaluate the predictive ability of four cytokines that had already shown promising results to confirm their potential to identify future INR.

In the context of HIV infection, it is common for IL-6, MIP-1β, and IP-10 plasma levels to be elevated in most PWH. A study by Jiao Y et al. examined the behavior of 26 cytokines, including IL-6, MIP-1β, and IP-10. Among these, only IP-10 consistently showed an association with HIV disease progression (based on CD4^+^ T-cell counts) during the acute HIV infection phase (12). However, other studies have reported contradictory results. One of them found no difference in IP-10 levels between IR and INR, but it did observe notable differences in IL-6 levels (9). Furthermore, Hernández-Walias et al. reported that PWH with poor immune recovery exhibited higher IL-6 levels than those with good immune recovery (15), but we did not observe significant differences between both groups. Furthermore, Prebensen et al. linked elevated plasma MIP-1β levels before starting ART with discordant long-term immune responses (10). However, we also found no difference between INR and IR in MIP-1β levels. Regarding PTX-3, Lee et al. identified a threshold of 1250 pg/mL, above which PWH exhibited poor immune recovery compared to those below this threshold. Our results also showed higher PTX-3 levels in INR, with a somewhat higher cut-off point (≥2173.5); however, statistical significance was not reached (11).

In conclusion, our findings suggest that IL-6, IP-10, MIP-1β, and PTX-3 are not good predictive markers for poor subsequent immune recovery.
